# On predictions and laws in biological evolution: Is biology able to formulate general laws and develop inductive predictions as in physics or chemistry?

**DOI:** 10.15252/embr.202154392

**Published:** 2022-03-14

**Authors:** Valentí Rull

**Affiliations:** ^1^ Botanic institute of Barcelona (IBB‐CSIC) Barcelona Spain

**Keywords:** Evolution & Ecology, History & Philosophy of Science

## Abstract

Biology has so far had difficulties formulating general laws akin to physics and chemistry. Evolution and its propensity to reduce entropy could become a start for such laws in biology.

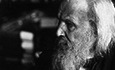

Science uses evidence‐based inductive reasoning to build theories, principles, and laws. A common type of inductive reasoning is generalization, that is, projecting conclusions drawn from one or a few case studies onto a broader context. The reliability of generalizations depends upon the representativeness and the formal validation of the selected case studies, which is usually performed by hypothesis testing. Another usual type of inductive reasoning is prediction, which uses observations to develop general principles and laws that can predict or anticipate future outcomes. The reliability of these predictions is confirmed by the accomplishment of the anticipated situation. It is interesting to note that generalizations are based on the analysis of empirical evidence, whereas predictions are formulated before the desired empirical evidence, which is actually the target of the prediction, is available.

… generalizations are based on the analysis of empirical evidence, whereas predictions are formulated before the desired empirical evidence, which is actually the target of the prediction, is available.

The American philosopher of science Peter Lipton (2005) commented that we are commonly more impressed by predictions than by accommodations, as he called hypothesis testing. To illustrate this, Lipton used the discovery of Halley's Comet. In 1705, the British astronomer Edmond Halley proposed that the comets observed in 1531, 1607, and 1682 were actually the same comet with a periodic elliptical orbit. Back then, his hypothesis did not have much impact within the scientific community. However, when Halley's prediction was confirmed in 1758 by the return of the comet, the intellectual world in Europe widely accepted the existence of a single comet, which was subsequently named Halley's Comet. Halley's prediction may seem straightforward, even trivial, considering the characteristic periodicity of 75 years in previous observations. Yet, it was the predictive success, rather than prior observations, that convinced the scientific community of his conclusion.

Physics is considered one of the strongest branches of science—along with chemistry and mathematics—in regard to the generality and accuracy of its predictions. Biology seems still to be in its infancy, and the search for regularities that could lend to potential generalizations is the most common approach (Dodds, 2009). This is due in part to the high level of complexity of the living world, its evolutionary change over time, and its relationships with the environment. As emphasized by the German evolutionary biologist Ernst Mayr (2004), these intrinsic and unique features of living beings, which are intimately associated with the genetic code, clearly differentiate biology from other natural sciences and make the fundamental laws of physics and chemistry insufficient to understand the living world.

The main aim of this essay is to discuss whether biological research is able to develop inductive predictions similar to physics or chemistry. First, I present some classical examples of physical and chemical discoveries based on inductive predictions, such as the Higgs boson, interstellar dark matter, and the periodic table of elements. As all these advances are based on the previous existence of fundamental laws, the question arises whether similar laws exist in biology to support physics‐like inductive predictions. I suggest that, if these laws exist, they should emerge from the evolutionary process, which is the main biological singularity. Thus, it should be possible to make inductive predictions based on the fossil record, which is the fundamental evolutionary evidence. Indeed, it seems that the lack of evolutionary laws is the main drawback for inductive prediction in studying evolution, which cannot escape to Lipton’s accommodation procedures, that is, hypothesis testing and generalization.

Physics is considered one of the strongest branches of science – along with chemistry and mathematics – in regard to the generality and accuracy of its predictions.

## Physical and chemical laws and predictions

The existence of the Higgs boson was predicted in the 1960s to fulfil the standard model of particle physics (SMPP) that describes the subatomic particles and three of the four fundamental forces—electromagnetic force, weak atomic force, and strong nuclear force. The equations of the SMPP accurately describe the electroweak force—that is, the combination of electromagnetic and weak forces—which is responsible for electricity, magnetism, light, and some types of radioactivity—assuming that the particles involved do not have mass, which is true for photons but not for particles such as W and Z bosons. This inconsistency was solved by proposing the existence of the Higgs field, named after the British physicist Peter Higgs, which would grant mass to any particle interacting with it. The particle associated with this field was tentatively called the Higgs boson, and its mass, charge, and spin were estimated with the SMPP. In 2012, after five decades of experiments, the Higgs particle was eventually discovered at the CERN Large Hadron Collider (LHC), and Peter Higgs, together with his Belgian colleague François Englert, was awarded the Nobel Prize in 2013 for this fundamental finding (https://home.cern/science/physics/higgs‐boson).

… physicochemical laws are viewed as immutable rules, and knowledge advances by finding the evidence needed to fulfill these laws.

Other physical predictions still await empirical confirmation, such as the existence of interstellar dark matter. Astrophysical observations of gravitational fields of galaxies are difficult to explain under gravitational laws, the fourth fundamental physical force, unless more matter is present than can be seen. This has led to the prediction that an unknown form of matter is abundant in the universe, which has been called dark because it does not interact with the electromagnetic field, that is, it does not absorb, reflect, or emit electromagnetic radiation and is therefore difficult to detect. However, dark matter particles are assumed to carry energy and momentum, and hence, they might theoretically be detectable in the LHC (https://home.cern/science/physics/dark‐matter).

**Figure 1:**
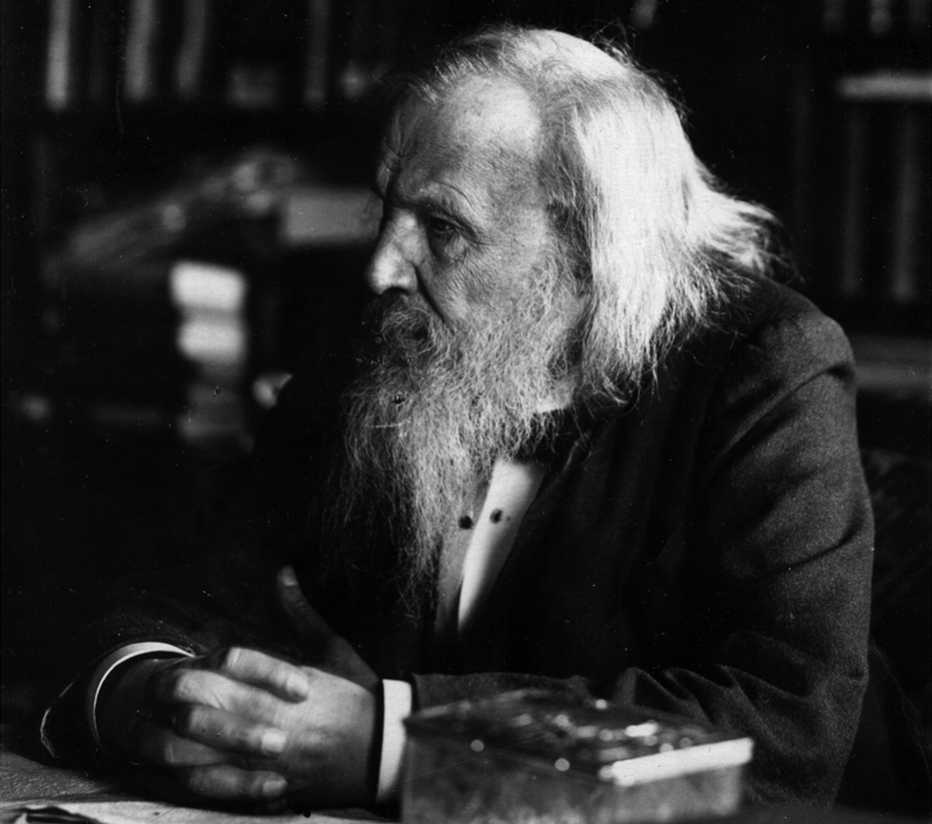
**Dmitry Mendeleev (1834–1907) Wikimedia/Public Domain**.

In chemistry, the periodic table is another example of successful predictive inference. The background behind this table is the periodic law, formulated in 1869 by the Russian chemist Dimitri Mendeleev (Fig 1), according to which the properties and atomic structures of the chemical elements are a periodic function of their atomic numbers (the number of protons in the nucleus), which is unique for each element. Mendeleev organized the chemical elements known at the time in a table according to their atomic numbers and observed gaps, which he considered to correspond to still unknown elements, and predicted the atomic composition of the unknown elements according to the periodic law. With time, these elements have progressively been identified to conform to the current periodic table.

A common feature of these predictions is the axiomatic nature of the fundamental background laws, in this case the SMPP, gravitational laws, and the periodic law of elements. Predictions of new subatomic particles, new chemical elements, or still undetected interstellar matter are necessary to prove these fundamental laws, and each time that a prediction is accomplished, it reinforces said laws. In other words, physicochemical laws are viewed as immutable rules, and knowledge advances by finding the evidence needed to fulfill these laws. A corollary is that the predictive approach seems better developed in scientific disciplines with well‐established fundamental laws.

## Biological laws

Among biologists is a lack of consensus whether general laws exist or not. Various scientists, including Mayr (2004), have contended that there are no laws in biology, owing to the contingent and unpredictable nature of living beings and their evolution. Others try to find regularities in biological contingency that could potentially inform general laws or they tentatively formulate biological laws, usually with a strong physical component and a recurrent emphasis on the laws of thermodynamics (Trevors & Saier, 2010; Brandon, 2013). However, fitting biology with the laws of thermodynamics cannot be considered a biological law; it is a physical law. In contrast, the propensity of evolution to minimize entropy (Rull, 2012), which presents a challenge to the universality of the second law of thermodynamics, may be the seed for developing a potential biological law. Notwithstanding, generally accepted laws specific to the biological world are still lacking. However, most theorists agree on a unique biological singularity: the diversity of living organisms and the complex spatial, temporal, functional, and ecological diversity patterns they generate emerge by evolution.

… the propensity of evolution to minimize entropy […] may be the seed for developing a potential biological law.

It follows that if general laws specific to biology do exist, they will likely derive from the theory of evolution. Thus, it is worth exploring its potential, along with recent developments in genomics and molecular phylogenetics, to support inductive predictions. Given the contingent nature of biological evolution (Mayr, 2004), predicting its future is still unworkable; but the possibility of making predictions similar to the Higgs boson, dark matter, or the lacking elements of the periodic table is worth a consideration. This endeavor should not be confused with a reductionist approach that aims to apply physical laws to biology; it is merely a conceptual and methodological comparison.

## Possible evolutionary predictions

Whether biological evolution progresses gradually or in leaps is an old debate. An example is the controversy between phyletic gradualism (PG) and punctuated equilibrium (PE). The PG concept proposes that evolution progresses slowly and gradually to transform one species into another (anagenesis) acting by natural selection on species' populations. In contrast, PE contends that most evolution takes place as rapid speciation events that split one species into two distinct species (cladogenesis), followed by long phases without significant evolutionary changes (stasis). The PE concept was proposed by the American paleontologists Gould and Eldredge (1977), who argued that gradual change is not observable in the fossil record, which is instead dominated by long‐ranging static fossil morphologies. The defenders of PG attributed these observations to the incompleteness and fragmentary nature of the fossil record.

The fossil record—similar to the first periodic table of Mendeleev, which was still incomplete—could be used as evidence to confirm either the PG or PE view. Under the PG rule, the fossil record should contain the whole range of intermediate morphologies that represent gradual anagenetic process. Furthermore, PG should eventually be able to predict these forms, as Mendeleev did with the hidden elements. There are three main limitations, however, that prevent such prediction: general or particular evolutionary laws of morphological change, similar to the periodic law of the elements, are lacking; fossil morphology represents usually the harder parts of a species and morphological changes in other, lost parts are unnoticed; and fossil morphology is just one of the possible phenotypic expressions of the genotype, which is the real evolutionary material. Even in the case that PG would be able to predict specific fossils, finding the necessary empirical evidence would be difficult owing to the intrinsic incompleteness of the morphological fossil record.

In contrast to PG, which is grounded in Darwin's theory of natural selection, PE is essentially based on the available morphological fossil record. Therefore, by definition, PE is comfortable with the fossil record as is and does not seem to have any predictions to do in this respect.

At present, the PG and PE proposals are considered two extreme views within the general context of evolutionary rates, which are not constant but variable across species (Futuyma, 2005). It is worth noting that our ability to accurately predict the fossils needed to fit with any evolutionary model, regardless of the involved rates, might provide the basis for formulating evolutionary laws and, therefore, for predicting future evolution. However, the limitations mentioned above seem insurmountable. The situation is therefore similar to the proposal of dark matter, as a huge amount of still hidden “dark evolutionary matter” is needed to properly understand biological evolution. The main difference is that physicists know what the target evidence is (the predicted dark matter particles) and the suitable methodology for finding it (particle acceleration), whereas biologists seem to ignore what we are looking for.

## The dark matter of evolution

Fortunately, our knowledge of the fossil record has greatly improved since the 1970s and 1980s, when the PG‐PE debate was rampant. Recent technologies to sequence ancient genome, or parts of it, preserved in fossil remains have added a large amount of dark evolutionary matter to the morphological fossil record. Importantly, the DNA of fossil organisms (also called ancient DNA or aDNA) is the evolutionary subject and, hence, the fundamental evolutionary matter. This technology inaugurated the so‐called field of paleogenomics, which may be able to reconstruct evolutionary trends, that is, the genetic changes in specific species over time. In spite of preservation constraints, paleogenomics has already provided direct insights into evolution that few would have predicted less than a decade ago (Cappellini *et al*, 2018). However, this particular field of knowledge progresses by accumulation of empirical evidence, rather than by its prediction, as in the case of the missing elements of the periodic table or interstellar black matter.

**Figure 2:**
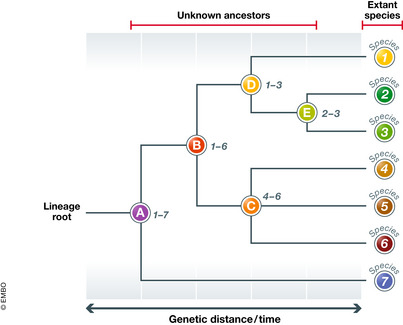
Hypothetical phylogenetic tree for seven imaginary extant species (1–7) The horizontal dimension can be expressed in genetic distance or in time units (usually millions of years before present), if the genetic distance is properly calibrated. The tree predicts that five unknown ancestors (A–E) should have existed and provides information on their main genetic traits and their evolutionary chronology, which may constitute a guide to what to look for in the molecular (and possibly the morphological) fossil record.

Moreover, we can consider the genome of contemporary species as a compendium of their evolutionary history, similar to a miniature fossil record. Recent advances in DNA analysis have again allowed the detailed reconstruction of the phylogenetic history of many living beings, as DNA is the evolutionary material itself. This procedure is somewhat similar to the prediction of the Higgs boson in that we would be able to anticipate the genotypic and, eventually, the phenotypic features of still undiscovered predecessors or “evolutionary bosons” (Fig 2). It would help us to know what we are looking for in the fossil record, which could be useful for planning research, just as physicists adjust the parameters of the particle accelerators based on the physical properties of their predicted targets.

Some of the abovementioned drawbacks still apply though, notably the fragmentary nature of the fossil record and the problems associated with the phenotypic expression of the genotype. The main difference is that we would need a different evolutionary boson for each lineage and each time period, which would make the prediction largely empirical rather than theoretical. Indeed, the prediction of evolutionary predecessors is not based on a fundamental law but on phylogenetic reconstructions, which are probabilistic hypotheses that vary over time with methodological improvements (Hawkins, 2006).

In summary, the attempts to use physical‐like inductive predictions for evolution seem to be useless, mainly due to the lack of fundamental evolutionary laws, which is likely a consequence of the incompleteness of the fossil record, but also of the intrinsic contingency and unpredictability of the evolutionary process. Therefore, inductive procedures in biological evolution, and in biology in general, still rely on hypothesis testing and generalization. However, some flaws in the hypothesis testing procedures persist and should be revised for improving generalizations.

## Persisting flaws of hypothesis testing

Some epistemological flaws may transform a hypothesis into a ruling theory, that is, a theory that controls and directs further research, notwithstanding whether it is built on sound evidence or a premature explanation based on insufficient empirical data. Eventually, a ruling theory may turn into a paradigm around which research is organized, and block further progress based on alternative explanations. This bad practice was already noted by the North American geologist Thomas Chamberlin (1890) 130 years ago and it still survives until today. Chamberlin proposed an alternative framework called the multiple working hypotheses (MWH): to develop any possible explanation and every testable hypothesis in order to promote thoroughness, suggest lines of inquiry that might otherwise be overlooked and develop the habit of parallel and complex thought.

… a ruling theory may turn into a paradigm around which research is organized, and block further progress based on alternative explanations.

The MWH is the ideal framework for Popperian falsification, which states that a scientific hypothesis cannot be definitively proved because, sooner or later, an alternative hypothesis may appear that is as good or better to explain the observed phenomenon. Therefore, the only possible procedure is to prove that a given hypothesis is false then move on to another to do the same and so on (Popper, 1959). The North American physicist John Platt developed the strong inference method of hypothesis testing, which may be regarded as a combination of the MWH and Popperian falsification approaches. Strong inference requires scientists to look constantly for alternative hypotheses and devise experiments or observations to exclude them. In other words, researchers should permanently question themselves what kind of evidence would be needed to disprove their own hypotheses (Platt, 1964).

Under this framework, hypothesis testing is more than merely proving a single hypothesis, as it requires explicit falsification of the competing explanations. In addition, different hypotheses must not necessarily be contradictory and mutually excluding, as they could have complementary aspects and may be united in a single, more general, explanation. Using MWP and Popperian falsification under a strong inference framework produces more sound explanations, leading to more robust generalizations, which may be more suitable to eventually discover biological laws.

Another flaw is trying to formulate hypotheses as predictions; yet, including the term “predict” does not transform a hypothesis into an inductive prediction. For example, predictive modeling does not necessarily mean that models make reliable predictions; rather the models are the hypotheses to be tested, even if this is rarely acknowledged. If the model’s outcome fit with actual observations, it is considered robust enough to be generalized as predictive tool. Otherwise, the model needs to be adjusted to accommodate empirical observations, which is analogous to hypothesis testing. The difference with inductive predictions, as used in physics, is that physical laws are considered immutable and that lack of empirical evidence to support them is due to time and technological improvement, rather than intrinsic deficiencies of these fundamental laws.

## Prediction or accommodation?

There is nothing intrinsically good or bad in inductive prediction and accommodating generalization *per se*, and different scientific disciplines may have diverse procedures, depending on the nature of the part of the world they study. The differences between living beings and dead matter are not in the world of subatomic particles or elemental composition, as these domains and their dynamics are common to both, but in the emergent properties generated at higher organization levels. Biological evolution, which uniquely characterizes living organisms, is therefore only possible at the level of DNA and the translation of the genetic code into the phenotype. Then, the lower organization level which differentiates living from dead matter is the molecular level. Above is the organismic level, with emergent properties also unique to living beings, such as birth, reproduction, and death. Still above, there is the ecological level, characterized by the relationships between species' communities and between those communities and their environment.

Therefore, if fundamental biological laws do exist, they should be looked for at the organismic or ecological levels rather than in the world of particle physics or elementary chemistry. The difficulty of finding such laws may be a consequence of the structural and functional hypercomplexity of these higher organization levels. However, some remaining bad practices in hypothesis testing procedures—notably the frequent use of the ruling theory approach—may also delay the attainment of biological laws. To circumvent these flaws, it is recommended to use the multiple working hypothesis framework, along with Popperian falsification and strong inference methods.

… if fundamental biological laws do exist, they should be looked for at the organismic or ecological levels rather than in the world of particle physics or elementary chemistry.

When considering our planet as an element of astronomical systems of higher hierarchy, the phenomenon of life seems to be lost in physical forces again. Regardless of the possibility of extraterrestrial life, the universe is ruled by large‐scale physical laws, such as gravity or electromagnetic radiation. Therefore, life may be considered an intermediate phenomenon between the molecular and astronomical levels, for which fundamental laws are elusive, despite the efforts of biologists to find them and the efforts of physicists to develop a theory of everything (Hawking & Mlodinov, 2010) that would reduce the phenomenon of life to fundamental physical laws.

It could be asked why biologists need to imitate conceptual methodologies of other scientific disciplines such as physics, chemistry, or mathematics and compulsively seek fundamental laws. Why do we need to change the paths of biological research and transform it into a science based on inductive predictions, rather than on hypothesis testing and generalization? The advances in biological knowledge using these accommodating procedures are plentiful and evident. What if, after all, fundamental laws only work for submolecular and astronomical worlds and life is a disturbing anomaly in between?

## Supporting information



Review Process File
