# Supplementary material for: On predictions and laws in biological evolution: Is biology able to formulate general laws and develop inductive predictions as in physics or chemistry?
Source: EMBO Rep. 2022 Mar 14;23(4):e54392. doi: 10.15252/embr.202154392 (PMC8982576; doi:10.15252/embr.202154392)
Supplement: Supplementary file 1 — Review Process File [file EMBR-23-e54392-s001.pdf]

EMBO reports

On predictions and laws in biological evolution

Valentí Rull
DOI: 10.15252/embr.202154392

Corresponding author(s): Valentí Rull (vrull@csic.es)

Review Timeline:

Submission Date:	25th Nov 21
Editorial Decision:	2nd Dec 21
Revision Received:	3rd Dec 21
Accepted:	22nd Feb 22

Editor: Holger Breithaupt

Transaction Report:

This article was editorially reviewed.

(Note: With the exception of the correction of typographical or spelling errors that could be a source of ambiguity, letters and reports are not edited. Depending on transfer agreements, referee reports obtained elsewhere may or may not be included in this compilation. Referee reports are anonymous unless the Referee chooses to sign their reports.)
